# Primary human monocyte differentiation regulated by *Nigella sativa *pressed oil

**DOI:** 10.1186/1476-511X-10-216

**Published:** 2011-11-21

**Authors:** Mahaya C Mat, Azman S Mohamed, Shahrul S Hamid

**Affiliations:** 1 Department of Chemical Pathology, School of Medical Sciences, Universiti Sains Malaysia, 16510 KubangKerian, Kelantan, Malaysia; 2 Advanced Medical and Dental Institute, Universiti Sains Malaysia, Lot 1-8, PersiaranSeksyen 4/1, Bandar Putra Bertam, Kepala Batas 13200, Penang, Malaysia

## Abstract

**Background:**

Oxidized low density lipoprotein plays an important role in development of foam cells in atherosclerosis. The study was focused on regulation of primary human monocyte growth and CD11b expression in presence of *Nigella sativa *oil.

**Methods:**

Primary human monocytes were isolated from whole blood and grown at 37°C and 5% CO2 saturation for five days prior to treatment with *Nigella sativa *oil. The cells were plated and washed before treatment with ox-LDL (10 μg/ml) as positive control and combined treatment of ox-LDL (10 μg/ml) and (140 ng/ml) *Nigella sativa *oil. The growth progression was monitored every 24 hours for 3 days.

**Results:**

Macrophages showed reduced growth in comparison to monocytes 24 hours after treatment with *Nigella sativa o*il. The mean cell diameter was significantly different between untreated and treated condition in monocytes and macrophages (p < 0.001). Similarly, intracellular lipid accumulation was hindered in combined treatment with *Nigella sativa *oil. This was further supported by cell surface expression analysis, where CD11b was markedly reduced in cells treated with combination oxLDL and *Nigella sativa *oil compared to oxLDL alone. More cells differentiated into macrophage-like cells when monocytes were supplemented with oxidized LDL alone.

**Conclusions:**

The finding provides preliminary evidence on regulation of cell growth and differentiation in monocyte and monocyte-derived macrophages by *Nigella sativa *oil. Further investigations need to be conducted to explain its mechanism in human monocyte.

## Background

Coronary artery disease (CAD) has continued to be the leading cause for the world's morbidity and mortality. Similarly, Ministry of Health of Malaysia declared coronary artery disease as the leading cause of hospital admission and non-accidental deaths over the last decade [1]. Data summary from 1994 to 2001 showed heart disease accounted for 14% to 16% of the principal cause of death in government hospitals in Malaysia [2].

Atherosclerosis is closely related to development of coronary artery disease [3]. The earliest visible lesion of atherosclerosis is the fatty streak, an aggregation of cholesterol-loaded macrophages or foam cells within the arterial wall [4]. LDL oxidation has been proven to be associated with atherosclerosis and has been regarded as a key step in atherogenesis [5]. Level of circulating oxidized LDL was higher in patients with coronary heart disease [6]. Macrophages will continue the uptake of oxidized LDL via scavenger receptors and further accumulate intracellular cholesterol before transforming into foam cells [7]. Differentiation of monocyte renders the cell ready for active participation in inflammatory and immune responses [8].

Macrophages are key players in many aspects of human physiology and disease [9]. A hallmark of the development of atherosclerotic plaques is the prior and concurrent accumulation in the arterial intima of lipoprotein particles subject to chemical modifications. This is associated with local inflammation in the vessel wall and further recruitment of monocytes from the circulation. By taking up such modified LDL (oxidized or acetylated), monocyte-derived macrophages are turned into fat-loaded macrophages residing in the vessel wall and furthering the local inflammatory response. The mechanisms underlying such foam cell generation has for several years been the focus of intensive research [10-14].

An experiment where a culture of monocytes with oxidized LDL, resulted in increased expression of CD14, TLR-4 (p < 0.001), there were as well as increased production of inflammatory mediators such as IL-6, IL-1β, and at a lower extent, TNF-α and MCP-1 factors [15]. It was evidenced that there was progressive increase in cellular size, density, granulation and expression of surface markers CD11b and CD36 during macrophage maturation [16]. In un-stimulated monocytes and granulocytes, CD11b are present in an intracellular, vesicular compartment, as well as on the cell surface. Inflammatory mediators were found to stimulate a 5 to 10 fold increase in Mac-1 (CD11b) and p150,95 on the cell surface [17,18]. Oxidized LDL and minimally modified LDL increased CD11b expression, suggesting the role CD11b for enhanced monocyte adhesion and this was demonstrated by prevention of monocyte adhesion by using anti-CD11b mAb [19].

The development of natural product has been drawing attention towards this treatment modality. Numerous studies have addressed the potential use of natural products. In Southern Asia and Middle East Asia for centuries, *Nigella sativa *L seed has been used to treat numerous diseases. More than 25% of drugs used during the last 20 years are directly derived from plants, while the other 25% are chemically altered natural products [20]. Studies have shown that the seed and oil exert very low degree of toxicity [21]. The chemical composition include 32-40% fixed oil, 0.4-0.45%% volatile oil, proteins, alkaloids, coumarins, saponins, minerals, carbohydrate and fiber [22-24]. The volatile oil contain 18.4-24% thymoquinone and 46% of monoterpenes such as ρ-cymene and α-piene [23]. El-Bahai *et al*. reported evidence of physiological and beneficial cardiac hypertrophy in rats induced by long term *Nigella sativa *supplementation [25]. There are also studies that have shown its promising anti-cancer effects in animal model [24]. Recent data directly implicate signaling by TLR4 in the pathogenesis of atherosclerosis, establishing a key link between atherosclerosis and defense against both foreign pathogens and endogenously generated inflammatory ligands [26]. The critical role of modified LDL in atherogenesis suggests the need to control its progression. Therefore, modulatory effect of *Nigella sativa *oil on the progression of monocyte-derived macrophage growth was investigated.

*Nigella sativa *was found to exerts its effects via suppressing productions of IL-6, TNF-α and NO, key pro-inflammatory mediators by primary macrophages, as shown in an experiment done using *Nigella sativa *aqueous extract [27]. *Nigella sativa *contains thymoquinone which has been reported as a potent anti-inflammatory and cytoprotective agent [28]. It also has been documented that thymoquinone is major bioactive component of *Nigella sativa *to upregulate expression of hepatocyte LDL receptor gene and suppress activation of HMG-CoA receptor [29]. However, it was found that thymoquinone-rich fraction extract showed higher activity compared to pure thymoquinone due to multiple bioactive compounds that work synergistically [30].

## Results

### Isolation and identification of primary human monocytes

**Figure 1:**
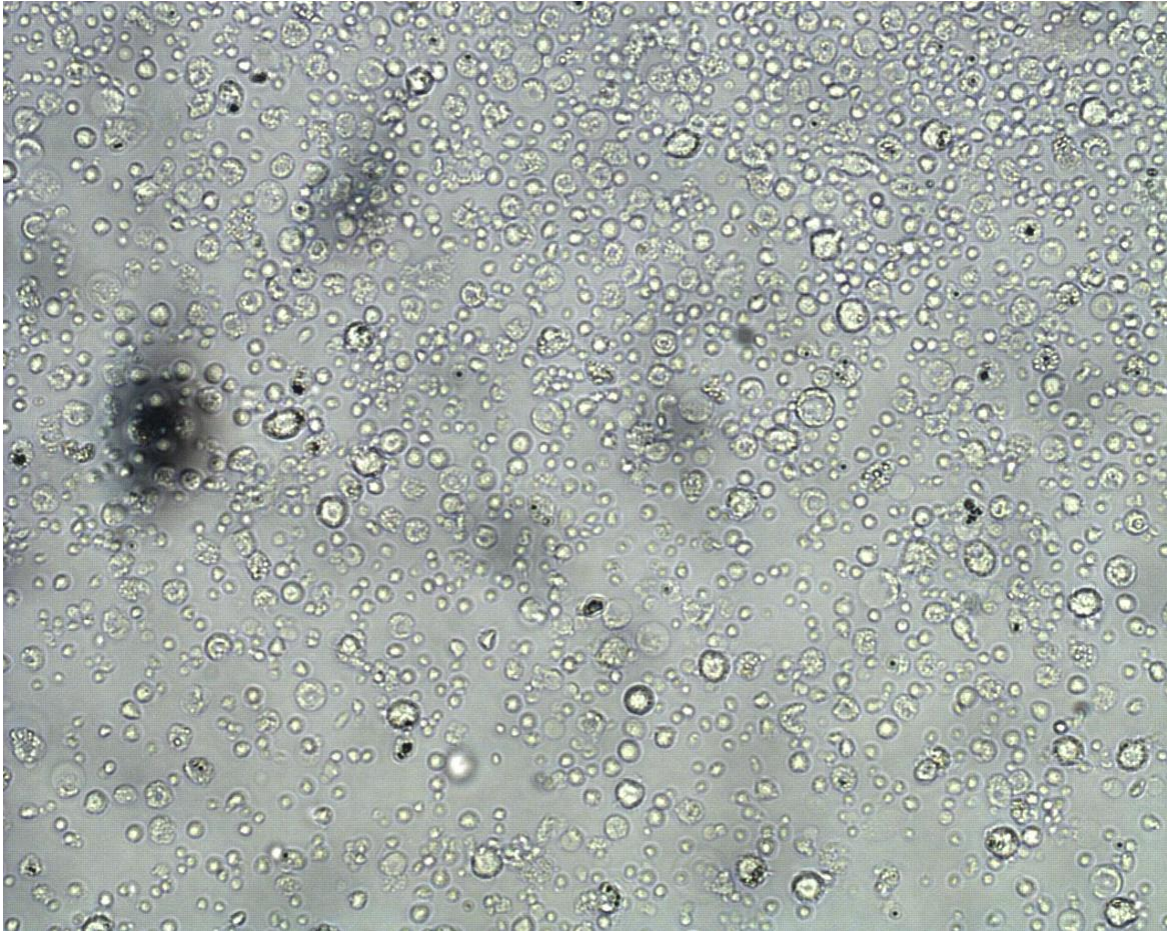
**Primary monocytes following isolation using magnetic Dynabeads under 100× phase-contrast microscope**.

**Figure 2:**
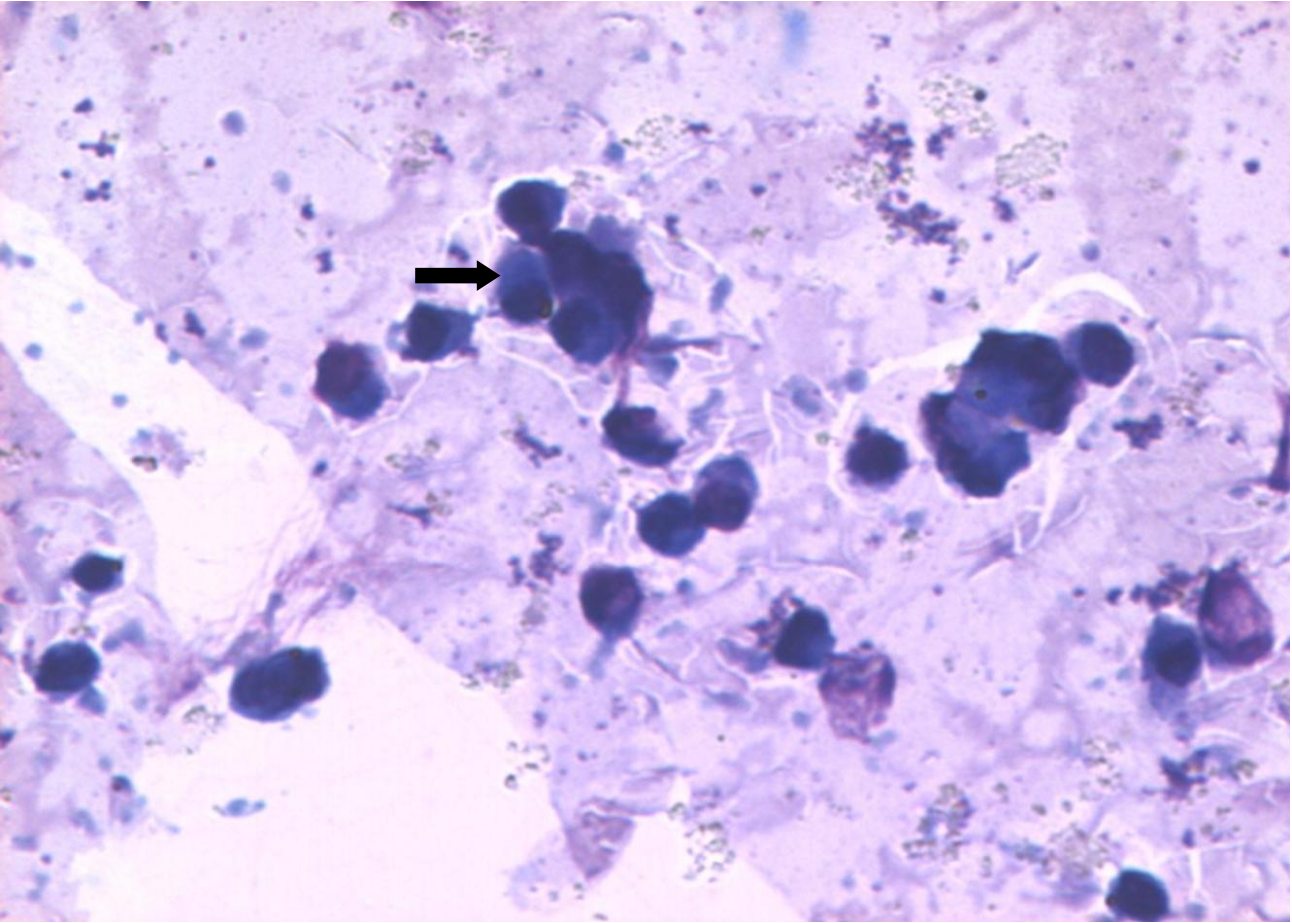
**The arrow indicates primary monocytes stained with Wright's stain visualized under 400 × light microscope**.

The cells isolated using MyPureDynabeadswere cultured in RPMI 1640 medium for growth. Images of primary monocytes isolated were captured using phase contrast microscope Axiovert 25 (ZEISS) as shown in Figure 1. The isolated monocytes were identified using Wright's stain smear and visualized under light microscope at 400× magnification. The cells were stained in blue and had kidney-shaped single nucleus with light blue shaded cytoplasm. Figure 2 shows monocyte under light microscope at 400× magnification.

**Figure 3:**
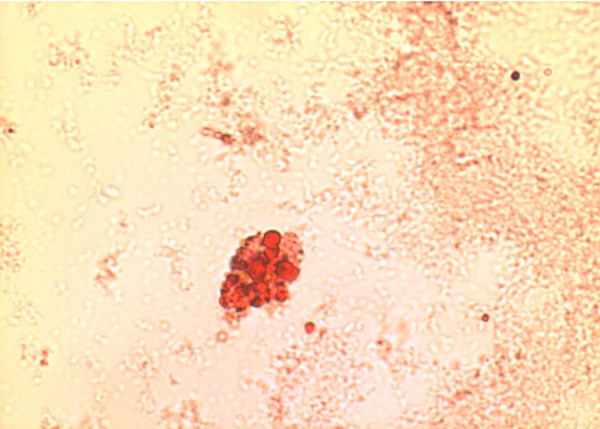
**Monocytes-derived macrophages at day 3 post-treatment with oxidized LDL, stained with oil red O stain and visualized under 400 × light microscope**.

**Figure 4:**
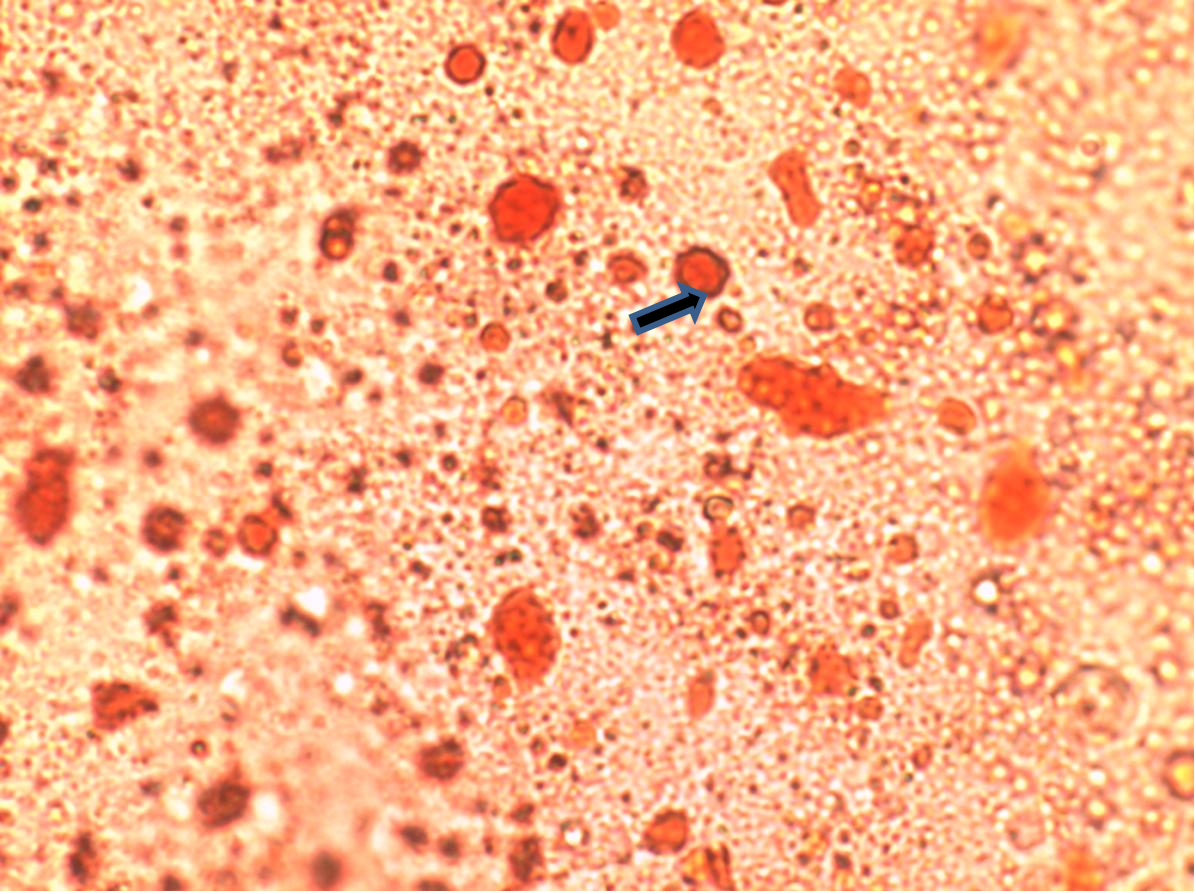
**Monocyte (arrow) supplemented with oxLDL and *Nigella sativa *oil 3 days post-treatment stained with oil red O stain and visualized under 400 × light microscope**.

**Figure 5:**
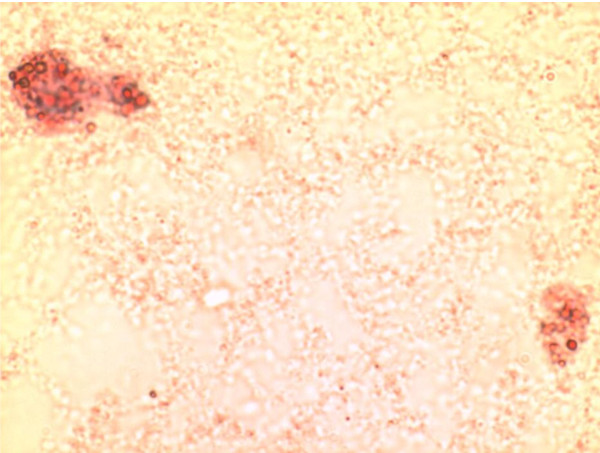
**Control primary monocytes-derived macrophage left 3 days untreated visualized under 400 × light microscope**.

The oil red O stain was used to identify vesicles that contain mainly cholesterol esters in the primary monocytes. Comparison of lipid accumulation between untreated cells and treated cells showed differences in the staining area. Figure 3 showed positive staining in monocytes following treatment with oxidized LDL. However, addition of *Nigella sativa *oil led to inhibited cell growth and impaired accumulation of oxidized LDL evidenced by the reduced intracellular staining in treated cells (Figure 4). The cells that were not exposed to either oxidized LDL or combination of oxidized LDL and *Nigella sativa *oil were seen to grow larger than those treated with combination of oxidized LDL and *Nigella sativa *but had small vesicles stained with oil red O (Figure 5).

### Percentage of Nigella sativa oil dilution

From the trypan blue dye cell exclusion method, the percentage of viable cells was more than 90% with 140 ng/ml Nigella sativa oil. The IC_50 _value obtained in this study was 180 ng/ml.

### Effects of Nigella sativa on oxidized LDL uptake by monocytes and macrophages

**Figure 6:**
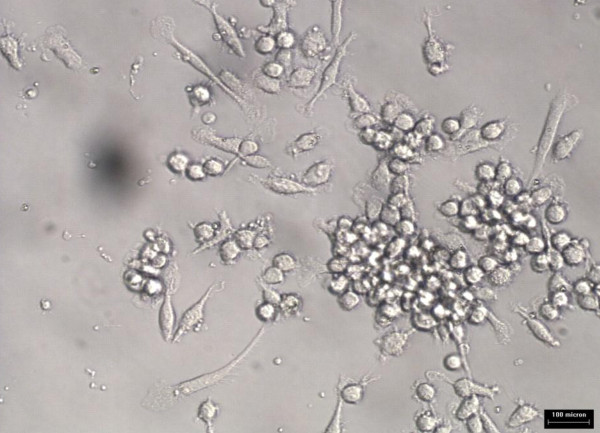
**Monocytes/macrophages supplemented with oxidized LDL at 24 hours**.

**Figure 7:**
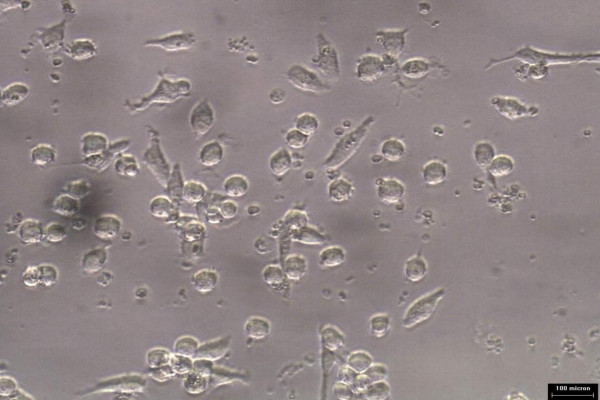
**Monocytes/macrophages supplemented with oxidized LDL at 48 hours**.

**Figure 8:**
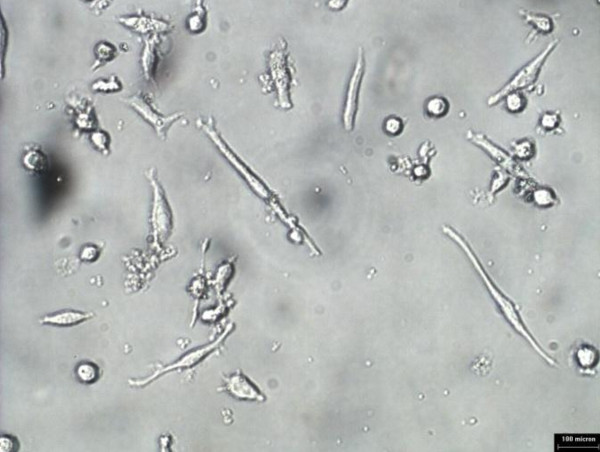
**Monocytes/macrophages supplemented with oxidized LDL at 72 hours**.

**Figure 9:**
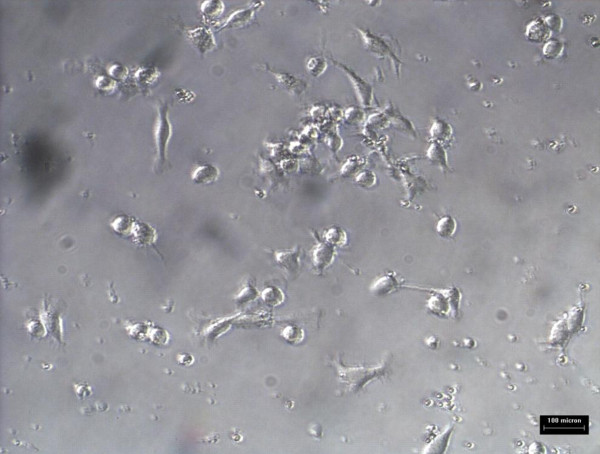
**Monocytes/macrophages supplemented with oxidized LDL and *Nigella sativa *oil at 24 hours**.

**Figure 10:**
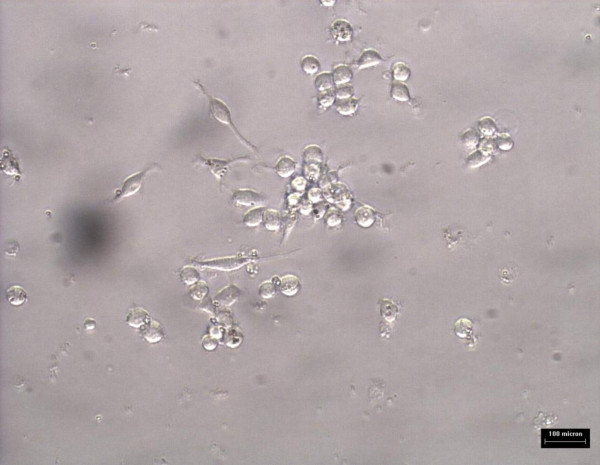
**Monocytes/macrophages supplemented with oxidized LDL and *Nigella sativa *oil at 48 hours**.

**Figure 11:**
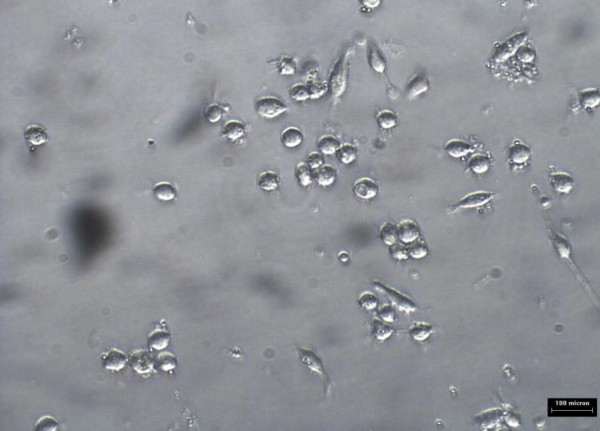
**Monocytes/macrophages supplemented with oxidized LDL and *Nigella sativa *oil at 72 hours**.

Monocytes/macrophages were grown in RPMI media for 5 days prior to addition of 10 ug/ml oxLDL or combination of 10 μg/ml oxidized LDL with 140 ng/ml *Nigella sativa *oil. The cells used as control untreated and treatment involved supplementation of oxidized LDL and *Nigella sativa *oil. These cells were left to grow in 5% CO_2 _at 37°C. The untreated cell growth was captured using 40× phase-contrast microscope at every 24 hours interval for 3 days as shown in Figure 6, 7, and 8, respectively. Images of treated cells were captured at 24 hours interval for 3 days as shown in Figure 9, 10, and 11.

The observation on cell growth showed both monocytes and macrophages were larger in size in the presence of oxidized LDL alone for 3 days compared to the combined treatment with *Nigella sativa *oil and oxidized LDL. There were more cells that differentiated into macrophage-like cells in monocytes supplemented with oxidized LDL alone.

**Figure 12:**
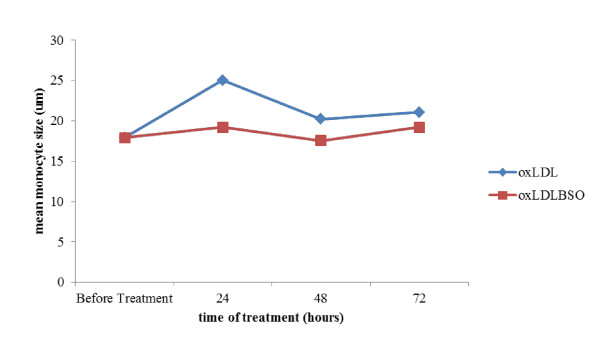
**Comparison on mean monocytes size within 3 days of treatment with 10 μg/ml oxidized LDL alone (oxLDL) and combination of 10 μg/ml oxidized LDL with 140 ng/ml *Nigella sativa *(oxLDLBSO) with timeline studied**.

**Figure 13:**
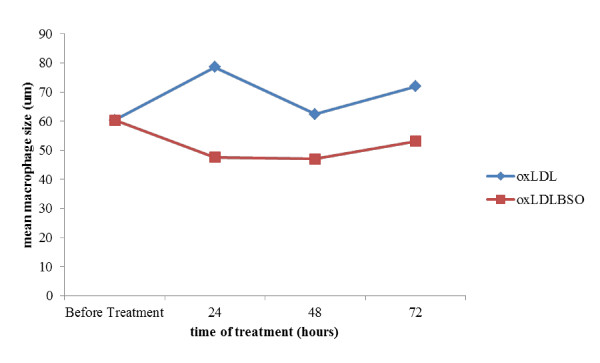
**Comparison on mean macrophages size within 3 days of treatment with 10 μg/ml oxidized LDL alone (oxLDL) and combination of 10 μg/ml oxidized LDL with 140 ng/ml *Nigella sativa *(oxLDLBSO) with timeline studied**.

Monocytes were found to be larger in diameter in presence of oxidized LDL throughout the timeline studied compared to the combined treatment of oxidized LDL and *Nigella sativa *(p < 0.001) (see additional file 1, Table 1). The similar findings were seen in macrophage diameter (p < 0.001) (see additional file 2, Table 2). Diameter differences in macrophage with combined treatment and treatment with oxidized LDL alone were 12.9% and 15.2%, respectively. This suggested there was reduced growth in macrophages upon exposure to *Nigella sativa *oil as shown in Figure 12 and 13. However, monocyte and macrophage showed gradual growth after 48 hours in combined treatment.

### Flow cytometry analysis of CD11b expression

**Figure 14:**
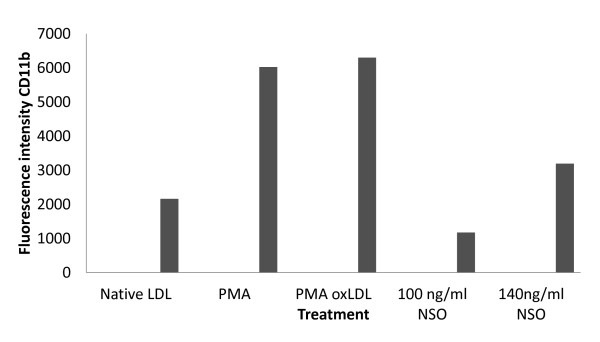
**Comparison of CD11b surface marker fluorescence intensity with treatment**. PMA = phorbol-myristate-acetate, LDL = low density lipoprotein, oxLDL = oxidized LDL.

Flow cytometry analysis showed the expression of surface marker CD11b fluorescence intensity of PE stained cells. The finding showed reduction of the fluorescence intensity in monocytes/macrophages treated with combination of *Nigella sativa *oil and oxidized LDL compared to cells treated with oxidized LDL alone. The differential expression was studied with 100 ng/ml and 140 ng/ml *Nigella sativa *oil treatment. It also included determination of surface antigen CD11b expression in cells supplemented with native LDL, PMA, PMA with oxidized LDL (oxLDL), 140 ng/ml and 100 ng/ml *Nigella sativa *were shown in Figure 14.

## Discussion

Medical plants or products have been related with anti-inflammatory properties in cell based studies [31,32]. Atherosclerosis is closely related to inflammatory process [33-35]. Among these medical plants is *Nigella sativa *L, that been reported to contain anti-oxidant and anti-inflammatory properties [36,37]. It acts by inhibiting COX and 5-LO pathways [36]. However, there is limited documentation on effects of *Nigella sativa *oil towards oxLDL intake in primary human monocyte.

We noted that *Nigella sativa *oil inhibited growth of monocyte and macrophage cells without causing toxicity. The number of viable cells was reduced to 50% when treated with 180 ng/ml *Nigella sativa *oil. Monocytes were found to be larger in size in the presence of oxidized LDL alone for 3 days compared to combined treatment with *Nigella sativa *and oxidized LDL. The similar finding was seen in macrophage growth. These findings were consistent with report by Yui *et al*. [38]. Oxidized LDL was able to induce the growth of murine macrophages *in vitro *[39]. However, cell growth was found to resume in both monocytes and macrophages after 48 hour. The declined inhibitory effect of *Nigella sativa *oil could be regulated by daily supplementation of the oil into media. Animal model studies reported daily dose of *Nigella sativa *caused profound effect in decreasing lipid peroxidation [40,41]. The effect of thymoquinone on proliferation of small cell lung cancer cells was shown to wane with time with less activity observed at 48 and 72 hours, suggesting more frequent dosing may be required to demonstrate a sustained effect [42]. This study showed 140 ng/ml *Nigella sativa *oil leads to reduced lipid accumulation within cells and decreased cell growth mainly macrophages within 48 hours treatment. This was in-line to an animal model study where rats were orally fed with *Nigella sativa *at concentrations of 50 mg/kg and 100 mg/kg body weight and resulted in reduction of lipid peroxidation [43]. It is still unclear whether the cell growth is regulated by expression of enzymes. Over expression of phospholipase A(2) lipolytic has been reported in macrophages, where it strongly increased foam cell formation upon incubation with native LDL and oxLDL [44].

The role of LDL oxidation has been claimed to be fundamental in the formation and progression of early atherosclerosis [45]. Human monocytes-derived macrophages esterified cholesterol from modified LDL more extensively than native LDL [46]. Oxidative modification of LDL can be induced by incubation with cell types like monocyte/macrophage and also other cell types such as endothelial cells and smooth muscle cells as reported by Steinberg [13]. Oxidized LDL is taken up rapidly by macrophages scavenger receptors [45]. A number of macrophage cell surface proteins have been identified which include class A scavenger receptor, CD36 and CD68. Fuhrman *et al*. also reported RAW264.7 murine macrophage cell line treatment with oxidized LDL caused inflammatory response by stimulating replication of monocyte-derived macrophages and entry of new monocyte into lesion [47].

The accumulation of cytoplasmic lipids droplets with oil red O staining in monocytic cells after exposure to oxidized LDL had been demonstrated [4]. We found treatment with combination of *Nigella sativa *and oxidized LDL showed no visible intracellular lipid vesicle and the monocyte surface was seen coated with oil red O. In addition, the morphological characteristics did not show significant development of monocyte into macrophage. The cells treated with oxidized LDL alone had intracellular stained lipid in several vesicles. Reduction of lipid stained deposits in combined treatment may be explained by presence of *Nigella sativa *oil. It may functionally block the oxidized LDL from inducing differentiation of monocyte into macrophages hence blocking the uptake by scavenger receptors. However, the actual mechanism on its action has to be further studied. The lipid deposits in aorta had also been observed in studies to see the effect of *Nigella sativa*. Animal model study indicated supplementation of either powder or oil form of *Nigella sativa *to hypercholesterolemic rabbits reduced lipid deposits of intimal surface of aorta indicating inhibition of plaque formation [48].

LDL can also be oxidized by incubation of LDL with a known catalyst of lipid peroxidation, such as copper or iron [49]. Copper was used in the oxidation of LDL in this study. Whereby, heavy oxidation of LDL up to 20 μg of protein/ml was used by Fuhrman *et al*. [47]. They reported that heavy oxidation of LDL was sufficient in inducing differentiation of monocyte into mature macrophages. It has been reported during monocyte to macrophage differentiation the expression of CD11b increased. Oxidized LDL triggers monocyte differentiation into macrophages and induced a distinct peak in fluorescence intensity and increased for sMFI for CD11b [19]. Activated monocytes in patients with active disease of Wegener's Granulomatosis were shown to have an increased surface expression of CD11b when compared to healthy control [50]. It was also shown that together with downregulation of CD11b, the ability of human blood monocytes to phagocytize opsonized *E. coli *also diminished [51]. These studies provide evidence on the importance of CD11b in the activation of monocytes. Fuhrman *et al*. found increased expression of CD11b of mouse peritoneal mononuclear cells isolated on 1,2 or 3 day after intra-peritoneal injection of oxidized LDL [47].

The flow cytometry data showed similar expression of cell surface marker, CD11b in monocytes/macrophages when treated with phorbolmyristate acetate (PMA) and oxidized LDL. PMA has been used as an artificial stimulator of monocyte-to-macrophage differentiation [52]. The native LDL did not induce growth of monocyte/macrophage culture. The cells were spherical and small as reported previously [53].

Differential expression of CD11b with certain doses of *Nigella sativa *explains its role in primary human monocyte-derived macrophage growth. This was shown by reduced CD 11b expression with 100 ng/ml *Nigella sativa *oil compared to 140 ng/ml. However, percentage of cell viability was similar in both these treatments. Further investigation would be carried out to identify the active ingredients found in *Nigella sativa *oil. It has been report the phytochemical content of Nigella sativa seed differ due to geographical and environmental factors mainly in its phenol and fatty acid contents [54]. Therefore, seeds sown in Malaysia would be characterized in terms of its phytochemical composition. This would provide precise understanding on regulation of monocyte differentiation by the active ingredients.

## Conclusions

Preliminary findings exhibited the presence of 100 ng/ml dilution of *Nigella sativa *oil caused downregulation in the differentiation of monocytes-to-macrophages based on phenotypic change and CD11b expression. The actual mechanism on how *Nigella sativa *oil exerts its anti-lipid and CD11b expression effects need to be further investigated.

## Methods

### Ethical Approval

The procedure was subject to approval by the University Ethical Committee. Written consent was obtained from healthy donors without medical history and without drug treatment. Approximately 50 ml of venous blood was collected into EDTA tubes using cannula fixed to vein.

### Primary human monocytes isolation

The primary cells were isolated using DynabeadsMyPure Monocyte Kit 2 for untouched human cells isolation kit (Invitrogen Dynal AS, Oslo, Norway). The cells were isolated from fresh whole blood collected from donor.

### Preparation of Buffer 1

Buffer 1 contained either 0.1% bovine serum albumin (BSA) or fetal calf serum (FCS) (Sigma-Aldrich, Germany) and 2 mM EDTA (Invitrogen, USA) in phosphate buffer saline (PBS) (Invitrogen GIBCO, New Zealand) at pH 7.4. The solution was filter-sterilized with a 0.2 μm membrane filter (WhatmanInc., USA) and stored at 4°C.

### Low Density Lipoprotein

Low density lipoprotein (LDL) was purchased from Milipore (Temecula, California, US). The LDL was isolated by ultracentrifugation method by Milipore and it was used in the study as a negative control.

### LDL Oxidation (Oxidized LDL)

The LDL was diluted in phosphate-buffered saline without EDTA (Invitrogen GIBCO pH 7.4 (1×) Auckland, New Zealand) to 100 μg/ml and dialyzed overnight against PBS at 4°C to remove the EDTA. LDL oxidation was done using an incubator shaker (Innova 4080) which was maintained at 37°C with 200 rotations per minute. LDL was incubated for 18 hours with the freshly prepared5 mM CuSO_4 _(BDH AnalaR). The oxidation was terminated by refrigeration at 4°C.

### *Nigella sativa *stock preparation and working solution

The pressed oil was produced by Baraka Oil Company in Sri Lanka. For the stock preparation, the oil was dissolved in 0.05% DMSO (Sigma Aldrich Co., USA). To make a preparation of 10 mL stock, 10 μL of Nigella sativa oil was mixed together with 5 μL DMSO. The 0.1% *Nigella sativa *stock solution was filter-sterilized using 0.2 μm membrane filter (Whatman Inc. USA) and stored at 4°C.

### Phorbol 12-Myristate13-Acetate (PMA) preparation

The Phorbol 12-Myristate13-Acetate (PMA) (Sigma, USA) was dissolved in DMSO and prepared to the concentration of 1 ng/ml. PMA. It was used in this study as a positive control.

### Culture Media Preparation

The RPMI 1640 (GIBCO) medium contained 1 mM L-glutamine (Sigma-Aldrich, USA), 200 U/mL pen-strep solution (Invitrogen GIBCO, New Zealand) and 10% FCS (Sigma-Aldrich, Germany). The FCS was heat-inactivated by preheating at 56°C for 30 minutes in waterbath. The media prepared were filter-sterilized with 0.2 μm membrane filter disc (Whatman Inc., USA) and stored at 4°C.

### RPMI 1640 Medium containing 5% Lipoprotein-deficient serum (LPDS)

This media was prepared using 1 mM L-glutamine (Sigma-Aldrich Inc., USA), 200 U/mL pen-strep (Invitrogen GIBCO, New Zealand) and 5% LPDS (Milipore, USA). The LPDS was used to replace FCS. The media were stored at 4°C after being filter-sterilized with 0.2 μm membrane filter disc (WhatmanInc., USA).

### Buffy coat preparation

The buffy coat was prepared from 50 ml of venous blood collected in EDTA tubes. The tubes were centrifuged at 800 × g for 20 minutes at room temperature (18-24°C) to obtain the separated layers of plasma, buffy coat and red cells. The supernatant was discarded and buffy coat was isolated from the EDTA tubes and transferred into sterile tube.

### Cell isolation using DynabeadsMyPure Monocyte for Untouched Human Cells

The monocytes were isolated using the DynabeadsMyPure Monocyte isolation kit. The procedure involved isolation of buffy coat from whole blood following centrifugation. A total of 10 to 18 ml of buffy coat was diluted in Buffer 2 to a final volume of 35 ml at 18 to 25°C at room temperature. The diluted buffy coat was added on top of 15 ml of lymphoprep (Axis-Shield, POC, Norway) and centrifuged at 160 × g for 20 minutes at room temperature. It was allowed to decelerate without brakes. Approximately 20 ml of supernatant was removed to eliminate platelets. Then it was centrifuged at 350 × g for 20 minutes at room temperature and allowed to decelerate without brakes. The MNC recovered from the plasma and lymphoprep interface was transferred to a 50 ml sterile tube. The MNC was washed once with buffer 1 by centrifugation at 400 × g for 8 minutes at 2-8°C and then washed twice with buffer 1 by centrifugation at 225 × g for 8 minutes. The MNC was suspended at 1 × 10^7 ^MNC per ml in Buffer 1. A total of 100 μl MNC in Buffer 1 was transferred to a tube and 20 μl of blocking reagent and antibody mix were added separately. This was mixed well and incubated for 20 minutes at 2-8°C. The cells were washed by adding 2 ml buffer 1 and mixed by tilting the tube several times before centrifugation at 300 × g for 8 minutes at 2-8°C. The supernatant was discarded and cells were suspended in 900 μl Buffer 1 (which was pre-cooled to 2-8°C). A volume of 100 μl of pre-washed Depletion MyOneDynabeads was added and mixed well before incubation for 15 minutes at 2-8°C by gentle tilting and rotation. The bead-bound cells were re-suspended by vigorous pipetting 5 times using a narrow tip opening. Then, 1 ml of pre-cooled buffer 1 was added and tube was place in the Dynal MPC-L (DynalMag 15) (Invitrogen, GIBCO, New Zealand) for 3 minutes. The supernatant was transferred to a new tube and placed again in the magnet for another 3 minutes. The supernatant obtained following these steps contained approximately 1 × 10^7 ^of untouched human monocytes.

### Culture growth

The platelet-free monocytes were resuspend in 5 ml RPMI 1640 supplemented with L-glutamine 1 mM, 200 U/ml penicillin-streptomycin and 5% heat-inactivated fetal calf or bovine serum. The cells were incubated in a humidified incubator with 5% CO_2 _and 95% air.

### Staining Methods

There are two types of cell staining methods were applied in the study. Wright's staining was done as confirmatory staining for monocytes. The oil red O stain was used to visualize cholesterol ester deposition in the monocytes.

### Wright's staining

The Wright stain was used for the air-dried smear. It produced the typical purple coloration of nuclei and neutrophilic granules. Methyl alcohol is used both as a solvent and fixative in this procedure. The nucleus and cytoplasm expanded after staining. The smear was immersed with Wright's solution for 4 minutes and followed by phosphate buffer solution for additional 9 minutes. Smear is then washed with water and air-dried. Finally, Cytoseal XYL was used for mounting of smear.

### Oil red O staining

Oil Red O is a lysochrome (fat-soluble dye) diazo dye used for staining of neutral triglycerides and lipids on frozen sections and some lipoproteins on paraffin sections. Oil Red O staining has to be performed on fresh samples as alcohol fixation removes the lipid. Oil Red O staining was used to view deposition of oxidized LDL in monocytes treated with oxidized LDL alone or oxidized LDL in the presence of *Nigella sativa *oil. The cell solution was air-dried on a slide before staining. A solution was prepared with 3 parts of 0.1% oil red O in isopropyl alcohol mixed with 2 parts of water. The slide was immersed into oil red O solution for 20 minutes and then dipped few times in 60% ethyl alcohol to clear the background. Staining was continued with Harris hematoxylin and then the slide was placed under running water. It was rinsed with distilled water and mounted with glycerin jelly. Fat component was stained deep red or deep orange, nuclei stained blue and other structures were unstained. The staining with Wright stain and oil red O stain for samples were done in duplicates.

### *Nigella sativa *oil toxicity

Cell viability was done using trypan blue dye (GIBCO/BRL) exclusion to determine the optimum concentration of *Nigella sativa *oil. The concentrations of *Nigella sativa *oil studied were 180 ng/ml, 140 ng/ml, 100 ng/ml, 70 ng/ml and 35 ng/ml. The number of viable cells was determined using hemacytometer. The cells were trypsinized before treatment with the trypan blue dye. A 20 μl of the suspension was collected and placed on a clean surface. Then, 20 μl of trypan blue dye (GIBCO, BRL) was added to the cell suspension and mixed before transferred to the edge of the haemocytometer slide and covered by a cleaned coverslip (The hemacytometer was earlier cleaned with 70% ethanol). The suspension mixture will automatically be drawn under the coverslip by capillarity. Any surplus fluid was then blotted and the slide was viewed under an inverted microscope. The cells were counted within the four corners of the grid. Both viable and non-viable cells were counted. The non-viable cells were stained blue. The total volume of the cells was then calculated using the formula given.

Cell counting formula:

$$\text{C}=\text{Av }\times 2^{*}\times 10^{4}\text{ cells}∕\text{ml}$$

Where; C = cell concentration (cell/ml)

Av = average number of cells in four corners counted

2* = dilution factor

Percentage of viable cells was counted as in the following formula:

$$\text{Percentage of viability}\left(\%\right)\;=\text{ Nv}∕\text{Nt}\times \text{1}00$$

Where; Nv = number of viable cells

Nt = number of cell population

### Uptake of oxidized lipoprotein

The cells were grown till confluence in RPMI 1640 media. The adherent cells were transferred from 25-ml culture flask and centrifuged at 220 × g for 8 minutes at 2-8°C. The cells were then plated in 24 well plate and supplemented with RMPI 1640 that contained 5% lipoprotein-deficient serum LPDS in replacement of FBS. Then cells were left to grow until complete adherence in humidified condition with 5% CO_2 _incubator at 37°C. The modulator effect of *Nigella sativa *on uptake of oxidized low density lipoprotein was studied by using 140 ng/ml of *Nigella sativa *oil. The study design comprised supplementation with oxidized LDL (10 μg/ml) alone and combination of oxidized LDL (10 μg/ml) and *Nigella sativa *oil (140 ng/ml). The microscopic images of the cells were taken at every 24 hours interval for 3 days.

### Microscopic analysis

The monocyte growth images were recorded using inverted phase-contrast microscope Axiovert 25 (ZEISS) pre-treatment, post-treatment day-1 and post-treatment day-5. The cells were stained using oil red O to visualize lipid engorgement into cells. The growth was determined based on cell diameter using Pro-plus software and type with positive staining of oil red O. Growth rate was determined based on difference in cell diameter before and after treatment.

### Flow cytometry analysis

Sample was prepared for cell surface antigen detection, CD11b using flow cytometry analysis by FACS Canto analyzer. The surface marker CD11b was detected using monoclonal antibody (BD Biosciences. San Jose36-37, Canada) reacted against CD11b antigen on monocyte surfaces. Analysis was done using software FACS Diva Version 6.1.2.

The sample preparation was done by isolating at least 1 × 10^4 ^cells into a 15 ml conical tube. The tube was centrifuged and media was removed to leave the cell pellets. The cells were then washed once with PBS at pH 7.4. This was followed by addition of 20 ug of CD11b monoclonal antibodies (BD Biosciences, Canada) into the tube and the tube was then incubated for 30 minutes in a dark place. After incubation the samples were washed again twice with PBS to remove excess antibody. The cells were then suspended in PBS for flow cytometry analysis.

Expression of surface marker CD11b was studied within 72 hours. The data obtained on 10,000 cells were stored in list-mode data files and analyzed by FACS Diva Version 6.1.2 software. The instrument was calibrated using CST Setup Beads (BD Biosciences, San Jose, CA). Monocyte subpopulation gating was based on forward scatter versus side scatter plot. To confirm the monocyte subpopulation, PE CD11b against side scatter was plotted.

The test samples included negative control (native LDL), positive controls (PMA, PMA with oxLDL), cells treated with 100 ng/ml dilution of *Nigella sativa *oil and treated with 140 ng/ml of *Nigella sativa *oil as shown in Figure 3, 4, 5, 6, 7, 8.

### Statistical analysis

Each experiment was done in triplicates. Analysis of results was done using t-test. All values were expressed as means ± standard deviation. Values of p < 0.05 were considered as significant.

## Competing interests

The authors declare that they have no competing interests.

## Authors' contributions

SSH and ASM were involved in the designing of the study. MCH and SSH participated in recruited of volunteer, isolation, identification and analyses. ASM contributed the antibody for flow cytometry and toxicity analysis. The manuscript was written by MCH. All authors have reviewed and approved the manuscript.

## Supplementary Material

Additional file 1 **Mean differences in monocyte growth**. (Results were expressed as mean ± s.d. p < 0.001 indicates statistically significant different. The experiments were done in triplicates). OxLDL = oxidized LDL, OxLDLNSO = oxidized LDL combined with NSO, Mean = mean diameter of cells in μm. Click here for file

Additional file 2 **Mean differences in macrophage growth**. (Results were expressed as mean ± s.d. p < 0.001 indicates statistically significant different. The experiments were done in triplicates). OxLDL = oxidized LDL, OxLDLNSO = oxidized LDL combined with NSO, Mean = mean diameter of cells in μm. Click here for file
