# Supplementary material for: Primary human monocyte differentiation regulated by Nigella sativa pressed oil
Source: Lipids Health Dis. 2011 Nov 21;10:216. doi: 10.1186/1476-511X-10-216 (PMC3280944; doi:10.1186/1476-511X-10-216)
Supplement: Additional file 1 — Mean differences in monocyte growth. (Results were expressed as mean ± s.d. p < 0.001 indicates statistically significant different. The experiments were done in triplicates). OxLDL = oxidized LDL, OxLDLNSO = oxidized LDL combined with NSO, Mean = mean diameter of cells in μm. [file 1476-511X-10-216-S1.DOC]

| Hours of treatment | Treatment | | Mean Differences  (95% Confidence Interval) | *p* value |
| --- | --- | --- | --- | --- |
|  | OxLDL  Mean (SD) | OxLDLNSO  Mean (SD) |  |  |
| 24 | 25.9 (15.24)  (n = 362) | 19.2 (7.09)  (n= 376) | 6.66  (4.88; 8.44) | <0.001 |
| 48 | 20.2 (3.44)  (n = 362) | 17.5 (2.71)  (n=378) | 2.67  (2.24; 3.11) | <0.001 |
| 72 | 21.0 (3.11)  (n=388) | 19.2 (2.81)  (n=383) | 1.85  (1.41; 2.30) | <0.001 |
