# Supplementary material for: Primary human monocyte differentiation regulated by Nigella sativa pressed oil
Source: Lipids Health Dis. 2011 Nov 21;10:216. doi: 10.1186/1476-511X-10-216 (PMC3280944; doi:10.1186/1476-511X-10-216)
Supplement: Additional file 2 — Mean differences in macrophage growth. (Results were expressed as mean ± s.d. p < 0.001 indicates statistically significant different. The experiments were done in triplicates). OxLDL = oxidized LDL, OxLDLNSO = oxidized LDL combined with NSO, Mean = mean diameter of cells in μm. [file 1476-511X-10-216-S2.DOC]

| Hours of treatment | Treatment | | Mean Differences  (95% Confidence Interval) | *p* value |
| --- | --- | --- | --- | --- |
|  | OxLDL  Mean (SD) | OxLDLNSO  Mean (SD) |  |  |
| 24 | 79.1 (64.20)  (n = 122) | 49.5 (35.89)  (n= 217) | 29.65  (18.38; 40.93) | <0.001 |
| 48 | 62.4 (27.50)  (n = 227) | 47.4 (20.05)  (n=328) | 15.03  (10.45; 19.60) | <0.001 |
| 72 | 72.9 (32.05)  (n=301) | 53.0 (21.18)  (n=209) | 19.98  (15.11; 24.85) | <0.001 |
